# Dual contraceptive method use and pregnancy intention among people living with HIV receiving HIV care at six hospitals in Thailand

**DOI:** 10.1186/s12978-016-0123-2

**Published:** 2016-02-03

**Authors:** Warangkana Munsakul, Rangsima Lolekha, Boonchai Kowadisaiburana, Anuvat Roongpisuthipong, Supannee Jirajariyavej, Suvanna Asavapiriyanont, Ubonsri Hancharoenkit, Benjamas Baipluthong, Sarika Pattanasin, Michael Martin

**Affiliations:** 1 Faculty of Medicine Vajira Hospital, Navamindharadhiraj University, Bangkok, Thailand; 2 Thailand Ministry of Public Health-U.S. Center for Disease Control and Prevention Collaboration, P.O. Box 139, Nonthaburi, 11000 Thailand; 3 Bamrasnaradura Infectious Disease Institute, Nonthaburi, Thailand; 4 Siriraj Hospital, Mahidol University, Bangkok, Thailand; 5 Taksin Hospital, Bangkok, Thailand; 6 Rajavithi Hospital, Bangkok, Thailand; 7 Wiang Pa Pao Hospital, Chiang Rai, Thailand; 8 Division of Global HIV/AIDS, Centers for Disease Control and Prevention, Atlanta, USA

**Keywords:** HIV infection, Pregnancy desire, Dual contraceptive use, Family planning, Thailand

## Abstract

**Background:**

Describe dual contraceptive method use and the intention to become pregnant of people living with HIV (PLHIV) and their partners in Thailand.

**Methods:**

From January 2008–March 2009, we systematically selected a cohort of PLHIV from PLHIV seeking care at five tertiary care hospitals and one community hospital to complete a questionnaire assessing sexual activity, intention to become pregnant, and contraceptive practices at baseline and 12 months after enrollment. Participants received short family planning messages every 2–3 months to promote the use of dual contraceptives and were offered family planning services.

**Results:**

A total of 1,388 PLHIV enrolled, their median age was 37 years (IQR 33–43), 898 (64.7 %) had a steady partner, and 737 (53.1 %) were male. Among those with a steady partner, 862 (96.0 %) did not intend to become pregnant; 709 (82.3 %) had sex during the previous 3 months, 683 (96.3 %) used at least one contraceptive method, and 202 (29.6 %) used dual contraceptive methods. Of the 317 PLHIV who used a single contraceptive method at baseline, 66 (20.8 %) reported using dual methods at 12 months. Participants at two tertiary care hospitals where coordinators facilitated PLHIV referral between HIV and OB/GYN clinics were more likely than participants at the other hospitals to change from single method to dual method (p ≤ 0.03).

**Conclusion:**

Few PLHIV in this study intended to become pregnant; however, only one-fourth used dual contraceptive methods. Integrating an assessment of the intention to become pregnant and strengthening the PLHIV referral systems in family planning services may contribute to higher rates of dual contraceptive use.

## Background

Approximately 450,000 adults were living with HIV/AIDS in Thailand in 2013 [1] and more than half of them were 18–49 years old. Thailand provides universal access to antiretroviral treatment (ART) under the national AIDS program and ART use has increased life expectancy and improved the quality of life of people living with HIV and AIDS (PLHIV) [2]. Longer life expectancy and an improved quality of life may increase the number of PLHIV who desire to become pregnant and have children [3]. A study in Canada showed that the proportion of HIV-infected women who desired children and intended to become pregnant increased once they were receiving ART [4].

Studies have shown that many PLHIV do not have access to family planning services [5–9]. Women with unintended pregnancies are more likely to have poor pregnancy outcomes (e.g., abortions, preterm birth, low birth weight) than women who plan their pregnancies [10]. HIV-infected women are also at risk of transmitting HIV to their infants and sexual partners. Thus, providing family planning services to PLHIV can improve the health of HIV-infected women and their children and reduce the risk of mother-to-child HIV transmission (MTCT).

The correct and consistent use of contraceptive methods is important to prevent unintended pregnancies and transmission of sexually transmitted infections (STIs) [11]. However, contraceptive methods vary in their effectiveness during routine use (i.e., including both incorrect and inconsistent use) and during perfect use (correct and consistent use) [12]. In PLHIV, the concurrent use of hormonal contraceptives and antiretroviral medications (the non-nucleoside reverse transcriptase inhibitors and protease inhibitors) can be associated with drug-drug interactions that may alter the contraceptive or antiretroviral effects of these medications [13]. In addition, contraceptives that are most effective at preventing pregnancy under routine use (e.g, hormonal contraceptives) provide no protection against HIV/STIs. Though condoms can prevent transmission of HIV and other STIs [5], inconsistent and incorrect condom use is common [12]. Studies report 14 to 21 % of people who use condoms alone become pregnant during the first year of routine condom use [12, 14]. As a result, the World Health Organization (WHO) recommends that PLHIV use dual contraceptive methods or dual protection to prevent unintended pregnancies and STIs [15]. Dual contraceptive method use is defined as the use of a barrier contraceptive (i.e., condoms), which can reduce transmission of many STIs, plus another effective family planning method that can prevent pregnancy as recommended by the World Health Organization (e.g., sterilization, hormonal methods, intrauterine devices, hormonal pills) [16, 17]. There are limited data on dual contraceptive use and the intention to become pregnant among PLHIV in Thailand. We assessed the intention of PLHIV to become pregnant and have children in the future and their use of dual contraceptive methods at six hospitals in Thailand.

## Methods

### Study population and procedures

From January 2008 to December 2009, every sixth PLHIV seeking care at HIV or Obstetrics and Gynecology (OB/GYN) clinics at four tertiary care hospitals in Bangkok, one in Nonthaburi, and one community hospital in Chiang Rai Province was offered enrollment in this cohort study. These hospitals were participating in the implementation and evaluation of a ‘prevention with positives (PwP)’ model. Information about the model has been presented elsewhere [18, 19]. The PwP model includes six prevention strategies: sexual and behavioral risk reduction, STI screening and treatment for PLHIV and their partners, promotion of HIV disclosure to partners, promotion of partner HIV testing, ARV treatment and adherence, and family planning and prevention of mother-to-child HIV transmission services. Health care providers including doctors, nurses, and counselors at the six hospitals were trained to deliver the PwP service model as part of routine service to all PLHIV attending the clinics. They provided the brief prevention messages [20] to all clinic attendees incorporating specific HIV prevention messages, based on needs of PLHIV at every clinical visit (usually every 3 months).

**Fig. 1:**
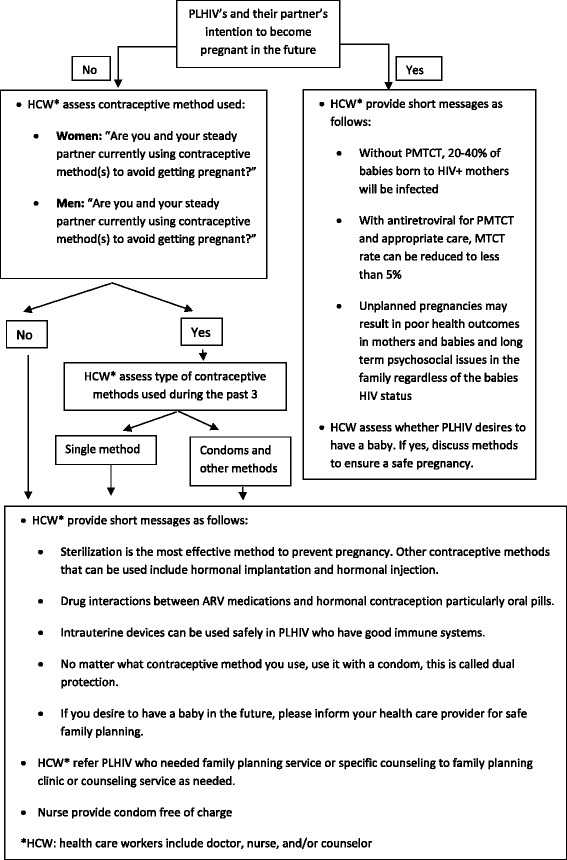
Pregnancy desire assessment and family planning short messages

In this study, we focused on the family planning strategy among PLHIV and their steady partners. A steady partner was defined as someone with whom the PLHIV had an emotional (e.g., loving, caring, respectful) and sexual relationship lasting at least 2 months. Assessment of pregnancy intention and needs for family planning, contraceptives, and short messages are shown in Fig. 1. PLHIV who requested family planning counseling or contraceptive service were referred for additional, more specific participant-centered family planning counseling or contraceptive service which were provided during routine clinical visits or, if necessary, scheduled for a date that was convenient for the health care worker and the PLHIV. The project hired a fulltime staff person for each of the two tertiary care hospitals (i.e., Siriraj and Rajavithi Hospitals) because there were insufficient staff at the two hospitals to complete study activities. The other hospitals were able to implement the PwP service model using existing staff.

Trained counselors provided PLHIV who were offered enrollment a brief description of study activities and PLHIV who wished to participate completed the informed consent process. PLHIV who wanted to enroll, signed the consent form. If a PLHIV did not want to participate, the next PLHIV presenting for care was offered the assessment. A trained counselor or nurse interviewed enrolled participants using a standardized questionnaire at baseline, and every 3 months for 4 visits (12 months ± 3 months) during a routine clinic visit to gather demographic data and information about their sexual activity and practices, disclosure of their HIV status to their partner, their partner’s HIV status, their partner’s and their intention to become pregnant in the future, and contraception practice during the previous 3 months. Contraceptive methods available to participants in the study included oral pills, hormonal injections, hormonal implants, male and female sterilization, condoms, and intrauterine devices. PLHIV received short family planning messages based on Thailand Ministry of Public Health (MOPH) Prevention with Positives Guidelines [20] (Fig. 1).

Counseling on sexual risk reduction, HIV disclosure, and ART adherence was provided as part of routine HIV care in the clinics and condoms were provided free of charge. PLHIV in this study were provided CD4 cell count and viral load (VL) testing free of charge by the National AIDS Program and ART if eligible according to Thai National Guidelines 2007 [21]. We abstracted CD4 cell count and VL test results (within 6 months) from patient medical records for analysis.

### Data analysis

Female PLHIV aged 18-49 years and males aged 18 years and older were selected for analysis at baseline. PLHIV who completed at least 4 visits including a visit at least 12 months after the baseline visit were included in the analysis. Data were analyzed and statistical tests performed using SAS 9.3 (SAS Institute Inc., North Carolina, USA). Pearson chi-squared and Fisher’s exact tests were used to test for differences in proportions. We used logistic regression to calculate the odds ratios (OR) of factors associated with using dual contraceptive methods at baseline and log-binomial regression to test for factors associated with changing from a single contraceptive method at baseline to dual methods at 12 months. Factors associated with changing to dual contraceptive methods in bivariate analysis (p-values <0.1) were included in a multivariable model. The analyses did not account for intra-cluster correlations by site since the sites were purposively selected from the six hospitals implementing PwP services. The effect of selecting groups of clients from clusters (i.e., hospitals) who may be similar in various aspects should be small in this study because clients were systematically sampled (every sixth PLHIV who visited the clinic) for assessment.

### Ethics approval

The study protocol, consents, and questionnaires were reviewed and approved by the Thailand MOPH Ethical Review Committee (ERC), Bangkok Metropolitan Administration (BMA) ERC, Siriraj Hospital ERC, and the U.S. Centers for Disease Control and Prevention Institutional Review Board.

## Results

### Baseline participant characteristics

From January 2008 to March 2009, 1,570 HIV-infected heterosexual men and women were recruited for the study. We excluded 137 women because they were pregnant and 45 female PLHIV who were 50 years or older from the analysis. Of the 1,388 PLHIV selected for data analysis, 737 (53.1 %) were male, their median age was 37 years (Interquartile range [IQR] 33–43 years) and 776 (55.9 %) were married or living with their partner. Participants had known their HIV status a median of 5 years (IQR 2–8 years). Most PLHIV (99.7 %) were taking ART and their median CD4 count was 343 (IQR 201–502) cells/mm^3^ and, among the 719 (51.8 %) who had viral load results, median viral load was <50 (IQR 40–50) copies/mL. Participant demographics characteristics are shown in Table 1.

**Table 1:** Baseline characteristics of participants in the assessment of family planning practices at 6 hospitals in Thailand, 2008-2009

Characteristics	Total (*N* = 1,388)	Male (*n* = 737; 53.1 %)	Female (*n* = 651; 46.9 %)
	No. (%)	No. (%)	No. (%)
Hospital			
Vajira (BKK)	202 (14.6)	102 (13.8)	100 (15.4)
Taksin (BKK)	165 (11.9)	99 (13.4)	66 (10.1)
Rajavithi (BKK)	93 (6.7)	29 (3.9)	64 (9.8)
Siriraj (BKK)	250 (18.0)	110 (14.9)	140 (21.5)
Bamrasnaradura Infectious Diseases Institute (Nonthaburi)	588 (42.4)	343 (46.5)	245 (37.6)
Viangpapao (Chiang Rai)	90 (6.5)	54 (7.3)	36 (5.5)
Age			
Median age in years (IQR)	37 (33, 43)	39 (34, 45)	35 (32, 40)
18–29 years old	164 (11.8)	69 (9.4)	95 (14.6)
30–39 years old	677 (48.8)	310 (42.1)	367 (56.4)
40–49 years old	447 (32.2)	258 (35.0)	189 (29.0)
> 50 years old	100 (7.2)	100 (13.6)	0 (0.0)
Education group			
≤ Primary school	533 (38.4)	257 (34.9)	276 (42.4)
> Primary school	853 (61.5)	478 (64.9)	375 (57.6)
Missing	2 (0.1)	2 (0.3)	0 (0.0)
Marital status			
Single	301 (21.7)	253 (34.3)	48 (7.4)
Married with and/or Live in partner	776 (55.9)	372 (50.5)	404 (62.1)
Divorced or Separated	311 (22.4)	112 (15.2)	199 (30.5)
Years since HIV diagnosis			
Median (IQR)	5 (2, 8)	5 (2, 7)	5 (2, 9)
< 1 year	120 (8.7)	61 (8.4)	59 (9.1)
1 to less than 5 years	529 (38.5)	298 (40.9)	231 (35.8)
≥ 5 years	726 (52.8)	370 (50.8)	356 (55.1)
Missing	13	8	5
On ART			
Yes	1,157 (99.7)	649 (99.7)	508 (99.8)
No	3 (0.3)	2 (0.3)	1 (0.2)
Missing	228	86	142
CD4 count at baseline^a^			
Median (IQR) cells/mm^b^	343 (201–502)	326 (181–475)	376 (225–530)
Viral load^c^			
Median (IQR) copies/mL	50 (40–50)	50 (40–50)	50 (40–50)
Received short messages relating to family planning at enrollment (PLHIV may receive messages from many staff)			
From doctor	927 (66.8)	514 (69.7)	413 (63.4)
From nurse	455 (32.8)	258 (35.0)	197 (30.3)
From counselor	1,042 (75.1)	571 (77.5)	471 (72.4)
Have steady partner			
No	490 (35.3)	290 (39.3)	200 (30.7)
Yes	898 (64.7)	447 (60.7)	451 (69.3)
Among PLHIV with steady partner (*n* = 898)			
Intention to become pregnant in the future			
Yes	36 (4.0)	13 (2.9)	23 (5.1)
No	862 (96.0)	434 (97.1)	428 (94.9)
Disclosed HIV status to steady partner	765 (85.2)	385 (86.1)	380 (84.3)
Steady partner HIV status			
Positive	385 (42.9)	183 (40.9)	201 (44.6)
Negative	288 (32.1)	161 (36.0)	127 (28.2)
Unknown	225 (25.1)	103 (23.0)	123 (27.3)
Among PLHIV with steady partner who had sex during the previous 3 months and had not had sterilization (*n* = 558)	558	291	267
Intention to become pregnant in the future			
Yes	34 (6.1)	13 (4.5)	21 (7.9)
No	524 (93.9)	278 (95.5)	246 (92.1)
Among 862 PLHIV not planning to become pregnant in the future			
Had sex during the previous 3 months	709 (82.3)	352 (81.1)	357 (83.4)
Contraceptive used during the past 3 months^b^	683 (96.3)	341 (96.9)	342 (95.8)
If contraceptive used (*n* = 683), methods used			
Single method used	481 (70.4)	257 (75.4)	224 (65.5)
Oral pills	12 (2.5)	4 (1.6)	8 (3.6)
Hormonal injection	4 (0.8)	0 (0.0)	4 (1.8)
Hormonal implantation	6 (1.2)	0 (0.0)	6 (1.2)
Sterilization	37 (7.7)	10 (3.9)	27 (12.0)
Condom	422 (87.7)	243 (94.6)	179 (79.9)
Intrauterine device	0 (0.0)	0 (0.0)	0 (0.0)
Two or more methods used^d^	202 (29.6)	84 (24.5)	118 (34.2)
Condom and sterilization	141 (69.8)	63 (75.0)	78 (66.1)
Condom and oral pills	36 (17.8)	13 (15.5)	23 (19.5)
Condom and hormonal implantation	16 (7.9)	3 (3.6)	16 (13.6)
Condom and hormonal injection	10 (5.0)	5 (6.0)	5 (4.2)
Condom and oral pills and hormonal injection	1 (0.5)	0 (0.0)	1 (0.8)

^a^Number of participants with CD4 count data = 1,200 persons

^b^Self or partner

^c^Number of participants with viral load data = 719 persons

^d^Pill/hormonal injection/hormonal implantation/male or female sterilization/condom/intrauterine device

Numbers might not sum to 100 % due to rounding

Of the 1,388 PLHIV who participated in the interview at baseline, 966 (69.6 %) participated in the interview at 12 months (4^th^ visit). Among the 422 PLHIV who did not participate in the interview at 12 months, 324 (76.8 %) were not interviewed because hospital staff were not available to conduct the interview according to the project schedule and 98 (23.2 %) declined to participate in the interview or were lost to follow-up for more than 180 days after an appointment.

### HIV disclosure to partner, partner HIV status, and pregnancy intent

At enrollment, 898 (64.7 %) PLHIV had a steady partner, 765 (85.2 %) had disclosed their HIV status to their partner, and 105 (34.7 %) of partners were HIV-infected.

Among the 898 participants who had a steady partner, 36 (4.0 %) reported they intended to become pregnant in the future. Excluding PLHIV and partners who had been sterilized, 34 (6.1 %) reported they intended to become pregnant in the future.

### Current contraceptive practices

Among 709 PLHIV who had no intention to become pregnant in the future and reported having sex during the previous 3 months, 683 (96.3 %) reported using at least one contraceptive method during the previous 3 months (Table 1). Only 202 (29.6 %) reported using two or more contraceptive methods (dual methods) and, in this group, 141 (69.8 %) used male or female sterilization and condoms. None of PLHIV in this study reported using intrauterine devices. Contraceptive methods used by participants are shown in Table 1.

### Changes in pregnancy intention and sexual behavior after receiving family planning short messages

Of the 898 PLHIV who had a partner, 623 (69.4 %) PLHIV came for follow-up at 12 months. Of these, 29 (4.7 %) reported at baseline that they intended to become pregnant, but 25 (86.2 %) changed their minds and reported they did not plan to have children at the 12 month visit. Only three of the 862 (0.3 %) PLHIV who did not plan to have children at baseline changed their minds and reported that they planned to become pregnant in the future at the 12 month visit. None of the PLHIV or their partners was pregnant at 12 months.

### Factors associated with dual contraceptive use at baseline

Factors associated with dual contraceptive use during the previous 3 months before baseline in multivariable analysis, included being female (adjusted Odds Ratio [aOR] 1.4; 95 % confidence interval [CI] 1.02–2.1), receiving care at Viangpapao Hospital (aOR 2.6; 95 % CI 1.3–5.0) or Rajavithi Hospital (aOR 2.6; 95 % CI 1.2–5.2), and being aware of their HIV status for 1 to 5 years (aOR 2.5; 95 % CI 1.1–5.5) or more than 5 years (aOR 2.6; 95 % CI 1.2–5.6) (Table 2).

**Table 2:** Factors associated with dual contraceptive use at baseline among PLHIV in the assessment of family planning practices at 6 hospitals in Thailand, 2008 − 2009

Characteristics	Use of dual methods (*n* = 683)		OR (95 % CI)	*p*-value	Adjusted OR (95 % CI)	*p*-value
	Yes (*n* = 202)	No (*n* = 481)				
	No. (%)	No. (%)				
Sex						
Female	118 (34.5)	224 (65.5)	1.6 (1.1–2.2)	<0.01	1.4 (1.02–2.1)	0.04
Male	84 (24.6)	257 (75.4)	1.0		1.0	
Age						
< 29 years	24 (24.5)	74 (75.5)	0.7 (0.3–1.8)	0.66		
30–39 years	110 (31.3)	241 (68.7)	1.0 (0.5–2.3)	0.94		
40–49 years	58 (28.9)	143 (71.1)	0.9 (0.4–2.1)	0.97		
> =50 years	10 (30.3)	23 (69.7)	1.0			
Education^a^						
< =Primary school	82 (31.1)	182 (68.9)	1.1 (0.8–1.6)	0.57		
> Primary school	120 (28.7)	298 (71.3)	1.0			
Marital status						
Single	2 (3.8)	51 (96.2)	0.3 (0.1–1.7)	0.17	0.3 (0.1–1.9)	0.22
Married/Live in partner	196 (32.9)	400 (67.1)	3.7 (1.3–10.6)	0.02	2.7 (0.9–8.2)	0.08
Divorce/Separated	4 (11.8)	30 (88.2)	1.0		1.0	
Time since HIV diagnosis^a^						
≥ 5 years	103 (30.5)	235 (69.5)	2.4 (1.2–4.9)	0.01	2.6 (1.2–5.6)	0.02
1 to less than 5 years	86 (31.3)	189 (68.7)	2.5 (1.2–5.1)	0.01	2.5 (1.1–5.5)	0.02
< 1 year	10 (15.4)	55 (84.6)	1.0		1.0	
Disclosure of HIV status to steady partner						
Yes	190 (31.8)	407 (68.2)	2.9 (1.5–5.4)	<0.01	1.7 (0.8–1.7)	0.14
No / No partner	12 (13.9)	74 (86.0)	1.0		1.0	
Steady partner HIV status						
Positive	105 (34.6)	198 (65.4)	1.5 (1.1–2.1)	0.01	1.2 (0.8–3.4)	0.32
Negative/unknown	97 (25.5)	283 (74.5)	1.0		1.0	
Hospital						
Viangpapao	22 (51.2)	21 (48.8)	3.5 (1.8–6.8)	<0.01	2.6 (1.3–5.0)	<0.01
Rajvithi	22 (43.1)	29 (56.9)	2.5 (1.4–4.7)	<0.01	2.6 (1.2–5.2)	0.01
Siriraj	29 (24.6)	89 (75.4)	1.1 (0.7–1.8)	0.72	0.8 (0.5–1.4)	0.55
BMA (Vajira&Taksin)	59 (35.5)	107 (64.5)	1.8 (1.2–2.8)	<0.01	1.5 (1.0–2.3)	0.08
Bamrasnaradura	70 (23.0)	235 (77.0)	1.0		1.0	
CD4^a^						
< 200	43 (33.1)	87 (66.9)	1.2 (0.8–1.8)	0.43		
> =200	133 (29.0)	326 (71.0)	1.0			
Ever received ART						
Yes	169 (30.9)	378 (69.1)	1.4 (0.9–2.1)	0.16		
Never	33 (24.3)	103 (75.7)	1.0			

Numbers might not sum to 100 % due to rounding

^a^Number may not equal to 683 due to missing value

### Factors associated with changing from a single contraceptive method at baseline to dual methods at 12 months

Of the 481 participants who reported using a single method of contraception during the previous 3 months at baseline, 317 (65.9 %) were re-interviewed 12 months later and 66 (20.8 %) had changed to use dual contraceptive methods. In the multivariable analysis, participants receiving care in Rajavithi (*p* = 0.03) and Siriraj (*p* = 0.02) Hospitals were more likely to change to dual methods than participants receiving care at the other sites (Table 3).

**Table 3:** Factors associated with changing from a single method of contraception at baseline to dual methods 12 months later among participants in the assessment of family planning practices at 6 hospitals in Thailand, 2008–2009

Characteristics	Use of dual methods at 12 months (*n* = 317)		RR (95 % CI)	*p*-value	Adjusted RR (95 % CI)	*p*-value
	Yes (*n* = 66)	No (*n* = 251)				
Sex^a^ (no.,%)						
Female	36 (19.0)	154 (81.0)	1.2 (0.8–1.9)	0.37		
Male	30 (23.8)	96 (76.2)	1.0			
Age (no.,%)						
< 29 years	12 (24.0)	38 (76.0)	1.1 (0.4–3.4)	0.87		
30–39 years	30 (18.7)	130 (81.3)	0.9 (0.3–2.5)	0.91		
40–49 years	21 (22.6)	72 (77.4)	1.0 (0.4–3.1)	0.80		
> =50 years	3 (21.4)	11 (78.6)	1.0			
Education^a^ (no.,%)						
< =Primary school	28 (20.6)	108 (79.4)	1.0 (0.6–1.5)	1.0		
> Primary school	38 (21.2)	141 (78.8)	1.0			
Marital status (no.,%)						
Single	0 (0.0)	27 (100.0)	N/A	0.34		
Married/Live in partner	65 (23.5)	211 (76.5)	3.3 (0.5–22.0)	0.20		
Divorce/Separated	1 (7.1)	13 (92.9)	1.0			
Disclosure of HIV status to steady partner (no.,%)						
Yes	55 (20.2)	217 (79.8)	0.8 (0.5–1.4)	0.65		
No / No partner	11 (24.4)	34 (75.6)	1.0			
Steady partner HIV status (no.,%)						
Positive	29 (20.6)	112 (79.4)	1.0 (0.6–1.5)	1.0		
Negative/unknown	37 (21.0)	139 (79.0)	1.0			
Hospital (no.,%)						
Viangpapao	4 (20.0)	16 (80.0)	1.5 (0.5–4.3)	0.48	1.5 (0.5–4.3)	0.43
Rajvithi	11 (47.8)	12 (52.2)	3.7 (1.9–7.2)	<0.001	2.5 (1.1–5.7)	0.03
Siriraj	26 (32.1)	55 (67.9)	2.5 (1.3–4.5)	<0.01	2.2 (1.1–4.2)	0.02
BMA (Vajira&Taksin)	13 (12.9)	88 (87.1)	1.0 (0.5–2.0)	1.0	1.0 (0.5–2.1)	0.98
Bamrasnaradura	12 (13.0)	80 (87.0)	1.0		1.0	
Time since HIV diagnosis at enrollment^a^ (no.,%)						
≥ 5 years	25 (16.9)	123 (83.1)	0.4 (0.2–0.8)	0.01	0.5 (0.2–1.0)	0.051
1–4 years	28 (20.4)	109 (79.6)	0.5 (0.3–0.9)	0.05	0.6 (0.3–1.1)	0.12
< 1 year	12 (38.7)	19 (61.3)	1.0		1.0	
CD4^a^ (no.,%)						
< 200	7 (20.6)	27 (79.4)	1.0 (0.5–2.1)	0.10		
> =200	54 (19.8)	219 (80.2)	1.0			
Ever received ARV^a^ (no.,%)						
Yes	45 (17.2)	217 (82.8)	0.5 (0.3–0.7)	<0.01	0.8 (0.4–1.6)	0.61
No	20 (37.0)	34 (63.0)	1.0		1.0	
Received short messages relating to family planning from nurse at enrollment (no.,%)						
No	25 (28.7)	62 (71.3)	1.6 (1.0–2.5)	.05	1.5 (0.9–2.4)	0.15
Yes	41 (17.8)	189 (82.2)	1.0		1.0	
Received short messages relating to family planning from doctor at enrollment (no.,%)						
No	41 (21.8)	147 (78.2)	1.1 (0.7–1.7)	0.70		
Yes	25 (19.4)	104 (80.6)	1.0			
Received short messages relating to family planning from counselor at enrollment (no.,%)						
No	42 (21.2)	156 (78.8)	1.0 (0.7–1.6)	0.94		
Yes	24 (20.2)	95 (79.8)	1.0			

Numbers might not sum to 100 % due to rounding

*RR* relative risk, *CI* confidence interval

^a^Number may not equal to 317 due to missing values

## Discussion

Most (96.3 %) PLHIV who reported having sex with their steady partner in our study used at least one contraceptive method but less than one-third used dual methods. The most common single method used was condoms (87.7 %). Because of inconsistent and incorrect condom use, condom use alone is associated with pregnancy rates as high as 18 % [15]. Therefore, dual contraceptive protection is strongly recommended to protect against HIV/AIDS and other STIs [13]. WHO urges healthcare providers to educate PLHIV on the benefits of dual protection use and to provide PLHIV access to contraceptives.

In this study, the most common combination of contraceptives used was condoms and sterilization (69.8 %). Our analyses indicate that female PLHIV and PLHIV who were aware of their HIV status for more than one year were more likely to use dual methods. This may be because PLHIV who were aware of their HIV status for more than one year were more likely to have received family planning messages and referral services as part of HIV service package. Women reported dual contraceptive use more frequently than men (*p* = 0.04). Women can independently initiate contraceptive choices, while men are not always aware of the partner’s contraceptive method and may not be aware of all contraceptives used (e.g., their partners’ oral or hormonal contraceptive use). A study in Chiang Mai, a Northern province of Thailand [22], showed that the rate of contraceptive use in postpartum HIV-uninfected women was high (97.6 %) and most used modern contraceptive methods; males reported condom use only 7.7 % of the time [22]. In contrast, a study of HIV-infected women in Chiang Mai found that 87 % of their partners used condoms [17, 23–25] and 18–56 % of the women used dual contraceptive methods; consistent with the findings of our study. Studies from India and Zambia have also reported that condoms and sterilization were the most common dual methods used among PLHIV in those countries [24, 25].

Studies have shown that integration of family planning services into HIV care is feasible and increases the use of contraceptives among HIV-infected women, and improves a variety of health and behavioral outcomes [12, 26]. In our study, the use of dual contraceptive methods varied by site (e.g., rate of dual methods was higher in Viengpapao Hospital and Rajavithi Hospital than Siriraj Hospital, Vajira Hospital, Taksin Hospital, and Bamrasnaradura Infectious Diseases Institute). None of the six sites provided one stop service of family planning service and HIV care. After we implemented short messages on family planning and referral services, one-fifth (20.8 %) of PLHIV who did not use dual contraceptive methods at baseline changed to dual methods at 12 months. This was particularly clear at Rajavithi and Siriraj Hospital, where there were project coordinators who helped facilitate referrals and link PLHIV to family planning services, whereas other hospitals implemented the services using routine staff and the routine referral system. Many HIV clinics in Thailand do not integrate family planning services with HIV care as a one stop service. Thus, having a staff person or a case manager who helps facilitate referrals and link PLHIV to family planning services can increase the likelihood PLHIV will access family planning services. Additional research on models that integrate family planning services in HIV clinics providing a one stop service would be helpful.

More than 80 % of reproductive age PLHIV who had a steady partner in our study reported having sex, but few (4.0 %) expressed a desire to have children. In contrast to studies in Europe and North America that report high fertility desire (69–75 %) and fertility intention (58 %) among reproductive age HIV-infected women [4, 26], our study found that only three HIV-infected women (0.3 %) who initially said they did not intend to become pregnant in the future because of their HIV diagnosis, changed their minds at 12 months. PLHIV in our study may not intend to become pregnant because more than half had not disclosed their HIV status to their partners and they may be concerned about discussing pregnancy and family planning with their partners. Furthermore, half of female PLHIV in our study were older than 35 years and may not want to become pregnant [27]. Other potential concerns among health care providers and PLHIV in Thailand include that PLHIV may transmit HIV to their infants, the misperception that HIV is an untreatable disease, and social discrimination that often occurs when others learn that one is infected [28, 29].

With access to ART free of charge under the Thai National HIV Treatment Program, HIV has become a manageable chronic disease. The Thailand National Prevention of Mother-to-Child HIV Transmission (PMTCT) Policy 2010 recommended using a lopinavir/ritonavir-based regimen for PMTCT [30] and the MTCT rate in Thailand was 2.3 % in 2012 [1]. Current evidence-based information and education should be provided to PLHIV and health care providers to help reduce stigma and discrimination.

The Thailand National HIV Treatment and Care Guidelines 2014 recommend that health care providers discuss pregnancy and childbearing intentions with all PLHIV, recommend effective and appropriate contraceptive methods (dual methods) to reduce the likelihood of unintended pregnancy and STI, provide reproductive options for HIV-concordant and serodiscordant couples who want to conceive (e.g., provide ART to the HIV-infected partner to have maximum viral suppression before attempting conception, provide assisted reproductive technology options, and consider administration of antiretroviral pre-exposure prophylaxis [PrEP] for HIV-uninfected partners) [31].

Our study had several limitations. Data were limited to PLHIV seen at five tertiary care hospitals in Bangkok, and one community hospital in the Chiang Rai province and PLHIV were on ART for average of 5 years; therefore, the results may not be generalizable to other settings in Thailand, to PLHIV who are not on ART, or to PLHIV who do not access to HIV prevention messages. Assessment of contraceptive methods used by men was challenging because some did not know their partners contraceptive method, resulting in an underestimate of contraception use in this study. Similarly, assessment of the intention to become pregnant of partners, particularly those who did not disclosed their HIV status, might be inaccurate. This model was implemented and integrated into routine hospital service during our evaluation, likely contributing to the 30 % of the PLHIV who did not participate in the interview at 12 months. This may have led to over or under estimates of some of the findings. About half of female PLHIV in our study were older than 35 years. Hence, the data in this survey may not accurately reflect the pregnancy intentions of younger reproductive age female PLHIV.

## Conclusion

In conclusion, less than one-third of PLHIV and their partners who did not intend to become pregnant used dual contraceptive methods. These findings highlight the need for assessing contraceptive methods used among PLHIV and their partners in routine clinical service. Our study showed that having a coordinator who can facilitate referral and link PLHIV to family planning services is important. In order to promote joint decision-making among couples for family planning, HIV disclosure to partners should be promoted. Future studies are needed to assessment pregnancy desire and intent and contraceptive use among young PLHIV. This is particularly important in Thailand as the treatment for all regardless of CD4 count policy [31] and getting to zero stigma and discrimination policy [32] are implemented, which will likely be associated with better treatment outcome and longer healthier lives for PLHIV. Other interventions that can help increase the use of dual contraceptive methods among PLHIV and their partners should be explored.
